# 3D Printed Monolithic Device for the Microfluidic Capture, Perfusion, and Analysis of Multicellular Spheroids

**DOI:** 10.3389/fmedt.2021.646441

**Published:** 2021-04-15

**Authors:** Alex Markoski, Ian Y. Wong, Jeffrey T. Borenstein

**Affiliations:** ^1^Department of Synthetic Biology and Bio Instrumentation, Draper, Bioengineering Division, Cambridge, MA, United States; ^2^Joint Program in Cancer Biology, Center for Biomedical Engineering, School of Engineering, Brown University, Providence, RI, United States

**Keywords:** microfluidics, cancer, 3D printing, spheroid, perfusion, monolithic

## Abstract

Microfluidic systems for the analysis of tissue models of cancer and other diseases are rapidly emerging, with an increasing recognition that perfusion is required to recapitulate critical aspects of the *in vivo* microenvironment. Here we report on the first application of 3D printing for the fabrication of monolithic devices suitable for capturing and imaging tumor spheroids under dynamic perfusion flow. Resolution of the printing process has been refined to a level sufficient to obtain high precision features that enable capture and retention of tumor spheroids in a perfusion flow stream that provides oxygen and nutrient requirements sufficient to sustain viability over several days. Use of 3D printing enables rapid design cycles, based on optimization of computational fluid dynamic analyses, much more rapidly than conventional techniques involving replica molding from photolithographic masters. Ultimately, these prototype design and fabrication approaches may be useful in generating highly multiplexed monolithic arrays capable of supporting rapid and efficient evaluation of therapeutic candidates in the cancer drug discovery process.

## Introduction

Additive manufacturing techniques such as 3D printing enable the construction of microfluidic devices with complex vertical features, including high aspect ratio pillars and channels that are challenging to fabricate using conventional techniques (1, 2). Historically, microfluidic devices have comprised planar structures that incorporate microscale channels for controlled manipulation of small fluid volumes (3). Typically, open channel geometries are molded as an elastomeric replica from a photolithographically-defined master mold, which is then adhered to a flat substrate to form a sealed microfluidic channel. The most commonly used substrate for replica molding, Poly(DiMethylSiloxane) (PDMS) (4), offers ease of use and high optical transparency but suffers from drawbacks such as chemisorption (5). Embossing techniques on hard plastics such as Cyclic Olefin Copolymer (COC) alleviate issues with sorption and mechanical instability, while retaining high optical clarity (6). However, microscale features can be easily distorted by differences in thermal expansion coefficient between the replica and substrate, as well as by mechanical deformation of the elastomeric substrate (7).Additional approaches, such as laser ablation of microchannels (8), extrusion-based micropatterned structures (9), and injection molding (10) have been reported for a range of microfluidic devices for lab-on-a-chip applications, with varying levels of resolution, surface roughness and optical quality (11).

Existing fabrication of microfluidic devices for cell and tissue culture often require laborious and time-consuming optimization of channel geometry, particularly when bonding together separate layers. Indeed, achieving strong adhesion between micromolded features and the substrate is challenging, particularly when the contact footprint is relatively small (12). Further, the incorporation of multiple layers as well as tubing connectors and bubble traps can add to the fabrication time and cost. 3D printing could facilitate rapid digital design and production cycles at considerably reduced cost and complexity. Previous work has utilized 3D printing to prepare master molds for replica molding of elastomeric materials, which still requires bonding to a substrate and remains limited to planar device geometries (2). Nozzle-based 3D printing has been used to directly print microfluidic devices, but typically exhibit large feature sizes governed by the diameter of ink droplets or extruded filaments, and have difficulty with overhanging features unless sacrificial support structures are included (1). Light-directed stereolithographic printing is advantageous since the lateral feature resolution is primarily governed by the pixel size of the illumination system, with increased mechanical cohesion across layers (1).

Multicellular spheroids are compact aggregates of cells that spontaneously self-assemble when cultured under low-adhesion conditions (13). These cell-dense constructs enhance cell-cell contacts, resulting in tissue-like barrier function and oxygen gradients, particularly in larger spheroids (diameter > 250 μm) (14). Thus, multicellular spheroids represent a promising *in vitro* model for pre-clinical testing of targeted therapies and immunotherapies on human cells (15). While three-dimensional constructs such as spheroids more closely recapitulate the physiological tumor microenvironment than do planar tissues, in static culture these 3D constructs experience sharp reductions in viability over periods longer than a few days, principally due to limitations in nutrient and oxygen diffusion. Spheroid culture within a microfluidic device permits active perfusion of oxygen and drugs, which extends viability significantly, but typically imposes significant additional design requirements (16, 17), described below.

First, spheroids must be trapped and held in place through strategically placed barriers within a microfluidic channel, thereby enabling maintenance of consistent flow conditions. A typical microfluidic trapping geometry is based on a “U” or “V” shaped array of pillars, which mechanically restrains spheroids above a certain size while permitting fluid to flow through the capture region. Second, spheroids must be perfused at a flow rate high enough to supply fresh media, but not so elevated as to reach damaging levels of fluid shear. Third, spheroids must remain mechanically intact after being introduced into the device and exposed to continuous perfusion. Finally, it may be desirable to permit spheroids to be analyzed using real-time methods such as optical imaging (i.e., confocal microscopy), and to be able to be retrieved for off-chip evaluation such as single cell analyses. Previously, a number of groups have used soft lithography to prepare PDMS microfluidic devices for spheroid culture (18–25). We and others have also demonstrated various designs for the capture and perfusion of intact biopsied tissue fragments from mice and humans (26–30).

In this brief report, we demonstrate 3D printing of monolithic microfluidic devices that integrate high aspect ratio pillars for spheroid trapping, as well as multilevel vertical features such as bubble traps and tubing connectors. We characterize these devices using finite element modeling to optimize flow conditions and capture efficiency. As a proof-of-concept, tumor spheroids were prepared and introduced into these devices and imaged with time-lapse fluorescence microscopy over periods of up to 3 days. The device reported here was capable of being printed and prepared for use within an hour and a half, constructed as a single unit with novel integrated connectors to minimize burdensome post-fabrication assembly. Within each channel, an array of 3D printed microposts was incorporated to capture and hold tumor spheroids in a perfusion flow stream for periods of several days, extending viability well-beyond what is typically observed for spheroids in static culture. Ultimately, these microfluidic devices will enable researchers and clinicians alike to test various cancer therapeutics such as immunotherapies in a scalable, rapid, and *in vivo*-relevant manner.

## Materials and Methods

### Materials

Photopolymerizable resin GR-1 was purchased from Pro3dure Medical GmbH (Dortmund, Germany). Perfluoroalkoxy tubing (1514L), polyetheretherketone tubing connector screws (XP-235X), and polyoxymethylene stopper screw (P-309) were purchased from IDEX Health Science LLC (Oak Harbor, WA, USA). Twenty milliliters sterile syringes (309661) were purchased from Becton Dickinson. A syringe pump (Standard Infuse/Withdraw PHD ULTRA) was purchased from Harvard Apparatus (Holliston, MA, USA).

CT26 colon cancer cells (CDL-2638), RPMI-1640 media, fetal bovine serum, and Trypsin EDTA solution 1X (30-2101) were purchased from ATCC. CellTracker Green CMFDA Dye (C7025), APC Annexin V (A35110), and UltraPure Agarose (16500500) were purchased from Invitrogen. PBS was purchased through ThermoFisher Scientific. 3D Petri Dishes (24–96, 24–35) were purchased from Microtissues, Inc.

### 3D Printing Resolution Testing

The Asiga Max X27 was used for all the light-directed 3D printed devices presented here. This printer uses a high power ultraviolet light emitting diode (385 nm wavelength) with digital light processing and has a pixel resolution of 27 by 27 μm. Computer aided designs were prepared in SolidWorks 2016 (Dassault Systems, Waltham MA) as an STL file at highest resolution (“fine”), then imported into Asiga Composer to optimize layer thickness and build orientation. Three types of test structures were printed to optimize feature resolution.

### Finite Element Modeling of Shear Conditions in Microfluidic Capture Region

Fluid flow and shear conditions in the microfluidic capture region were computationally modeled in COMSOL 5.3 Multiphysics (COMSOL, Inc., 100 District Avenue Burlington, MA, USA). The geometry was initialized from a top-down snapshot of the device design exported from SolidWorks 2016. This simulation solves for laminar flows based on the Navier-Stokes equation with boundary conditions of a 1 mm entrance length and thickness, with no-slip wall conditions, and assuming a fluid viscosity of 1 cP and flow rates of 5 uL/min. The mesh was set to a physics-controlled mesh setting, with normal element size with a minimum mesh quality measure of 0.1537 μm, chosen to be sufficiently small for modeling a characteristic feature size of 76 μm.

### Cell Culture and Spheroid Formation

CT26 cells were subcultured from 1:4 to 1:10 ratios with media replacement every 2–3 days, based on the recommended ATCC protocol. Prior to seeding, CT26 cells were trypsinized and stained with CellTracker Green CMFDA Dye (Invitrogen C7025) at a concentration of 20 μM in PBS for 15 min, then resuspended in media to a cell density of at least 1 × 10^6^/mL.

Molten agarose was cast against the 3D Petri Dish elastomeric masters, then allowed to cool and solidify. The cooled agarose molds were placed in a 12 well plate and prepared for the introduction of cells by soaking in 2.5 mL complete growth media twice for 15 min. To seed 200 μm diameter spheroids, the 24–96 3D Petri Dish geometry was used with a 75 μL droplet of containing 96,000 cells. To seed 300 or 500 μm diameter spheroids, the 24–35 3D Petri Dish geometry was used with a 75 μL droplet of containing 118,000 and 547,000 cells, respectively.

### Microfluidic Device Setup

A 5 mL syringe with a gauge 12 blunt tip was used to obtain the spheroids for transfer into the device's bubble trap port. Transfer into the channel was done by gently pushing the syringe's plunger with the syringe inside the bubble trap and angled toward the capture geometry. Validation of sample capture was completed using a fluorescence microscope for visual verification.

### Confocal Microscopy

A total of five CT26 spheroids were set up for imaging over a course of 3 days. Controls for this test consisted of a spheroid in a device without flow to evaluate the rate of cell death caused by the device. The loaded devices were placed in a Zeiss LSM 710 confocal microscope with 488 and 594 nm Argon lasers and environmentally controlled conditions (37°C and 5% CO_2_). Using Zen 2.3 Blue software, images were acquired every 600 min using a 10X objective (imaging 14 slices at 25 μm steps). Images were recorded under consistent acquisition parameters (e.g., exposure time, camera gain/gamma control, and microscope aperture).

Analysis of each set of images was done *via* the use of ImageJ (Rasband, W.S., ImageJ, U. S. National Institutes of Health, Bethesda, Maryland, USA, https://imagej.nih.gov/ij/, 1997–2018.). Mean gray values and integrated density functionalities values along with area of the outlined spheroid were used to calculate the corrected total cell fluorescence (CTCF) relative to a background region at the bottom of the image.


(1) $$\begin{array}{l} \text{CTCF}=\text{Integrated Density}-(\text{Area of selected cell}\\ \;\times \text{Mean fluorescence of background readings}) \end{array}$$

Corrected total cell fluorescence was used to calculate the percent decrease in brightness when compared between the values at 10 and 70 h. The 10 h time point was chosen as the baseline due to unintended inconsistencies in brightness associated with the 0 h images.

## Results

### Optimization of 3D Printed Feature Resolution

Light-directed 3D printing occurs through spatially selective photopatterning of successive layers. The vertical spatial resolution is primarily determined by the layer thickness, while the lateral spatial resolution of light-directed 3D printing is dependent on light exposure. Thus, underexposure results in uncured resin, while overexposure results in larger features than expected. Indeed, light exposure must be carefully optimized for patterning hollow microfluidic channels with an overhanging ceiling. Overexposure of the channel walls could result in narrower or occluded channel cross-sections, while underexposure of the ceiling could result in poor mechanical strength and collapse.

**Figure 1:**
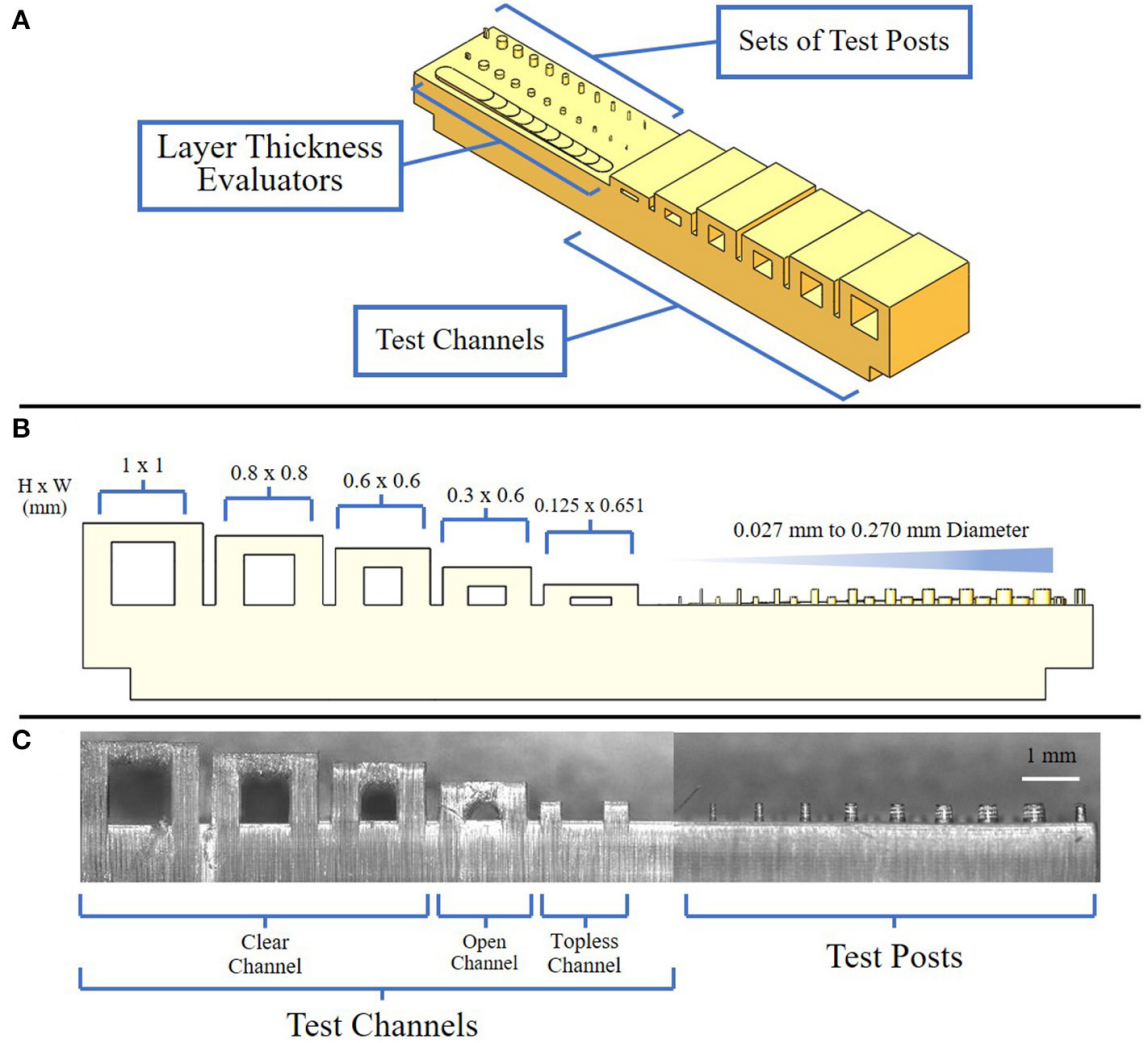
Views of resolution testing device. **(A)** Isometric view of the resolution testing device composed of three major components: test channels, test posts, and layer thickness evaluators; **(B)** Side view of resolution testing device highlighting expected channel and post-dimensions; **(C)** Side view of a resolution testing device showing examples of clean, open, and topless channels.

In order to optimize the printed features to achieve sub-100 μm resolution, we used a scanning white light interferometer to characterize the layer thickness using a series of steps designed with vertical offsets of 10 μm (Figure 1A). A vertical offset of 50 μm was determined to produce channel dimension features closest to the CAD model, based on multiple tests with sequential image analysis. Next, we designed a test structure pattern consisting of an array of cylindrical posts with diameters ranging from 27 to 270 μm at two different heights of 125 and 250 μm (Figure 1B). In addition, rectangular channels were created with dimensions ranging from 650 μm wide by 125 μm high as the smallest to 1 by 1 mm as the largest. For these features, the cure through multiplier (CTM) is defined as the cure thickness coefficient which is factored into the 3D printer's calculations when defining the over curing per layer. As the 3D printer cures each layer, it also cures past the assigned layer thickness, which helps ensure effective adhesion between each layer. The offset value, which is an unadjusted distance to cure through in conjunction with the CTM, determine the amount the printer cures into previous layers. We systematically varied the CTM from 0.1 to 2.0 and the offset value from 10 to 600 μm. An imbalance in CTM and offset value resulted in channels being clogged with cured resin due to over curing, the tops of channels not printing, and pillars with a diameter of 81 μm and below not retained on the platform during cleaning post-printing (Figure 1C). Based on these tests, we found that a CTM of 1.0 and offset value of 272 μm resulted in printed features most consistent with the CAD model. Once optimized, the feature resolution for the height and width of the channel when normalized with respect to the 3D CAD model showed a 2.1% (9.2 ± 8.2 μm) and 2.9% (22 ± 10 μm) deviation, respectively, resulting in a small increase in size compared to the CAD file. Overall, 100% of the optimized devices constructed were capable of sustaining perfusion for 70 h while capturing and holding spheroids of 300 μm in diameter.

### Microfluidic Device Design

**Figure 2:**
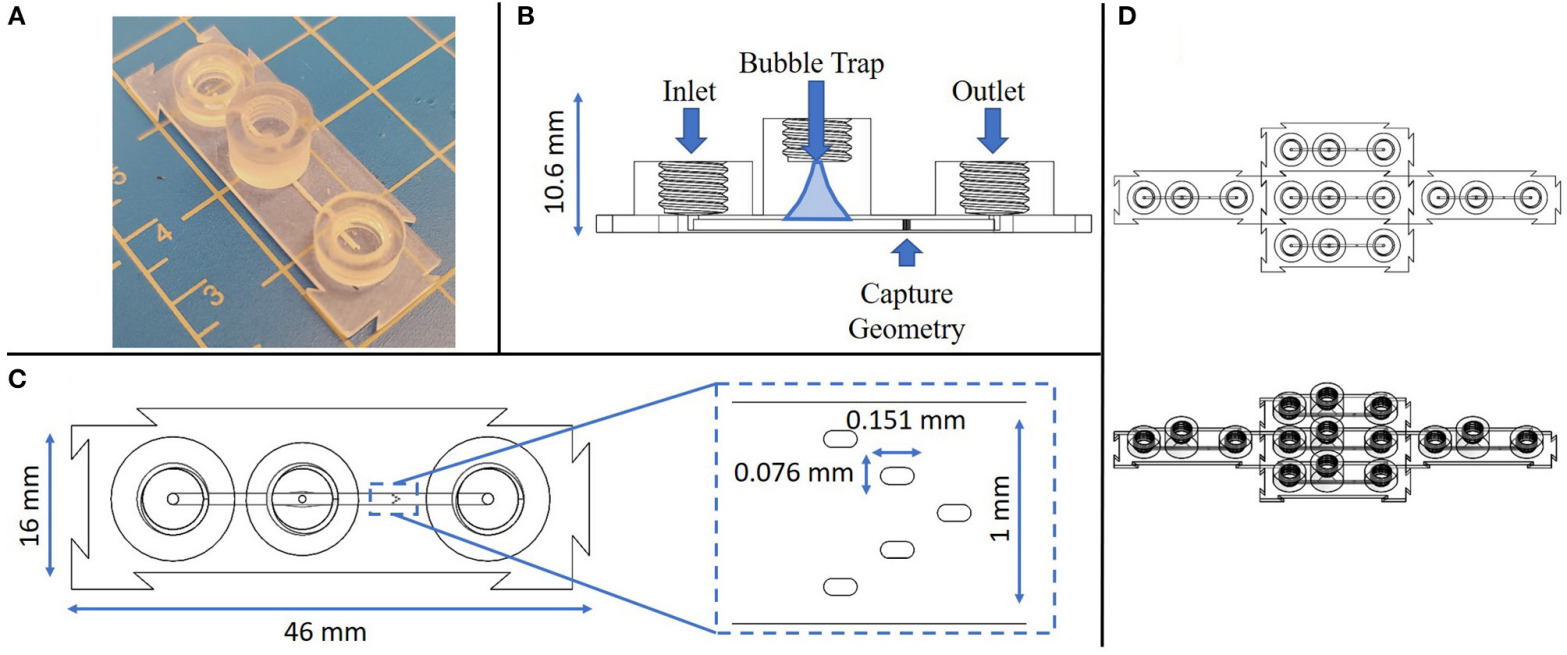
Multiple views of the monolithic spheroid perfusion device with annotations. **(A)** Isometric view of the device; **(B)** Cross-sectional view through the center of the device with labels for key features; **(C)** Top-down view of the device with an expanded view of the capture geometry; **(D)** Top down view of 5 replicate devices connected *via* interlocking features at the individual device edges.

The design and features of the 3D printed device improved on previous reports of tumor microenvironment structures (20, 21), in several significant ways, including the formation of high precision micropost arrays as capture features, as well as monolithic integration of printed fluidic connectors and bubble traps into a single construction step. The 3D printed device consisted of a single channel with an embedded spheroid capture feature, a bubble trap, and three threaded ports along the channel, on each for the inlet, bubble removal, and outlet, respectively (Figure 2A). A single straight channel was chosen for its simplicity, modifiability, and easily controlled flow characteristics (Figure 2B). The length of the straight channel design was chosen so that an injected spheroid would successfully reach the feature, since the distance is minimized between the injection port and the capture feature. Samples were observed to reach the capture feature within 30 s of being injected into the channel, and would settle into a fixed location within minutes.

Next, a bubble trap was designed to prevent any bubbles from reaching the capture geometry, by creating a tall curved chamber that allowed bubbles to rise up and out of the path of flow along the bottom of the chamber (Figure 2B). Any bubbles that entered the device would be directed toward the bubble trap where, due to their buoyancy, they would follow a path of the top of the channel up and out of the perfusion stream. The curvature of the chamber narrows in a parabolic manner toward a port at the top of the chamber, a location that can be used to remove any trapped bubbles. Removal of bubbles is accomplished by removing any connectors from the threaded port at the top of the bubble trap and letting flow push the bubbles out. The bubble trap reported here is smaller and more compact in size than in previous reports (20, 21).

The capture region is the most crucial feature of this design, since it must capture a biological sample without damaging it throughout the testing process. In prior reports, capture was achieved through the design of intersecting channels with opposing flow currents, along with a depressed feature to entrain a tissue in the flow stream (17). However, a high precision micropost array, similar to structures previously used for tissue capture in embossed and laminated thermoplastic devices (27–29), represents a superior means for maintaining perfusion flow with a firmly fixed tumor fragment or spheroid. Further, the microposts provide fiducial marks that can be used to precisely locate regions in the spheroid for monitoring cell viability and death in a spatiotemporal manner. Printing of this feature was optimized through multiple iterations to identify a design that would ensure capture without occluding the channel, physically damaging the sample upon contact, or preventing flow and nutrients from reaching the sample (see Supplementary Figure 1).

The micropost array design was derived based on several considerations, including prior reports (27, 28) the limits of the Asiga 3D printer feature resolution, and its adaptability to a variety of sample sizes (200–700 μm). The capture geometry consists of a series of five slot-shaped posts in a V-shaped formation oriented in a concave configuration in line with the channel flow, such that a biological sample would be captured and entrained by the surrounding posts (Figure 2C). Both computational modeling and tumor fragment loading experience has shown that the presence of sharp corners or angles in capture features leads to undesirable performance. In the case of fluid flow, sharp features such as rectangular or sharp-tipped posts lead to regions of disturbed flow, causing local variations in shear rates that may potentially damage delicate fragments and certainly lead to non-uniform exposure of the fragments to soluble factors such as immune checkpoint compounds in the flow stream. From the standpoint of tumor capture and entrainment, sharp or angular features have been seen to cut into fragments, increasing the likelihood that portions of the entrained samples may be sheared off in the flow stream. The closer the post size is to the captured fragment size, the less likely these shear-damage-related effects are to occur. We have found that the presence of five posts in a concave pattern represents the simplest design capable of gently but firmly entraining fragments in the flow stream (25).

An additional unique feature of this device is the modular nature of the connector geometry located around its perimeter. The goal of these modular connectors is to permit precise assembly of multiple components in an arrayed format, allow for any number of configurations to be achieved with this device, and to remove any restrictions on testing sample size or setup. As long as these devices have a flat surface to rest on, they are able to connect to each other on each side without restriction (Figure 2D). The connector geometry has a tolerance of ~200 μm, which allows for minimal difficulty when linking devices while ensuring as tight connection.

### Finite Element Modeling of Microfluidic Flow and Shear Conditions

**Figure 3:**
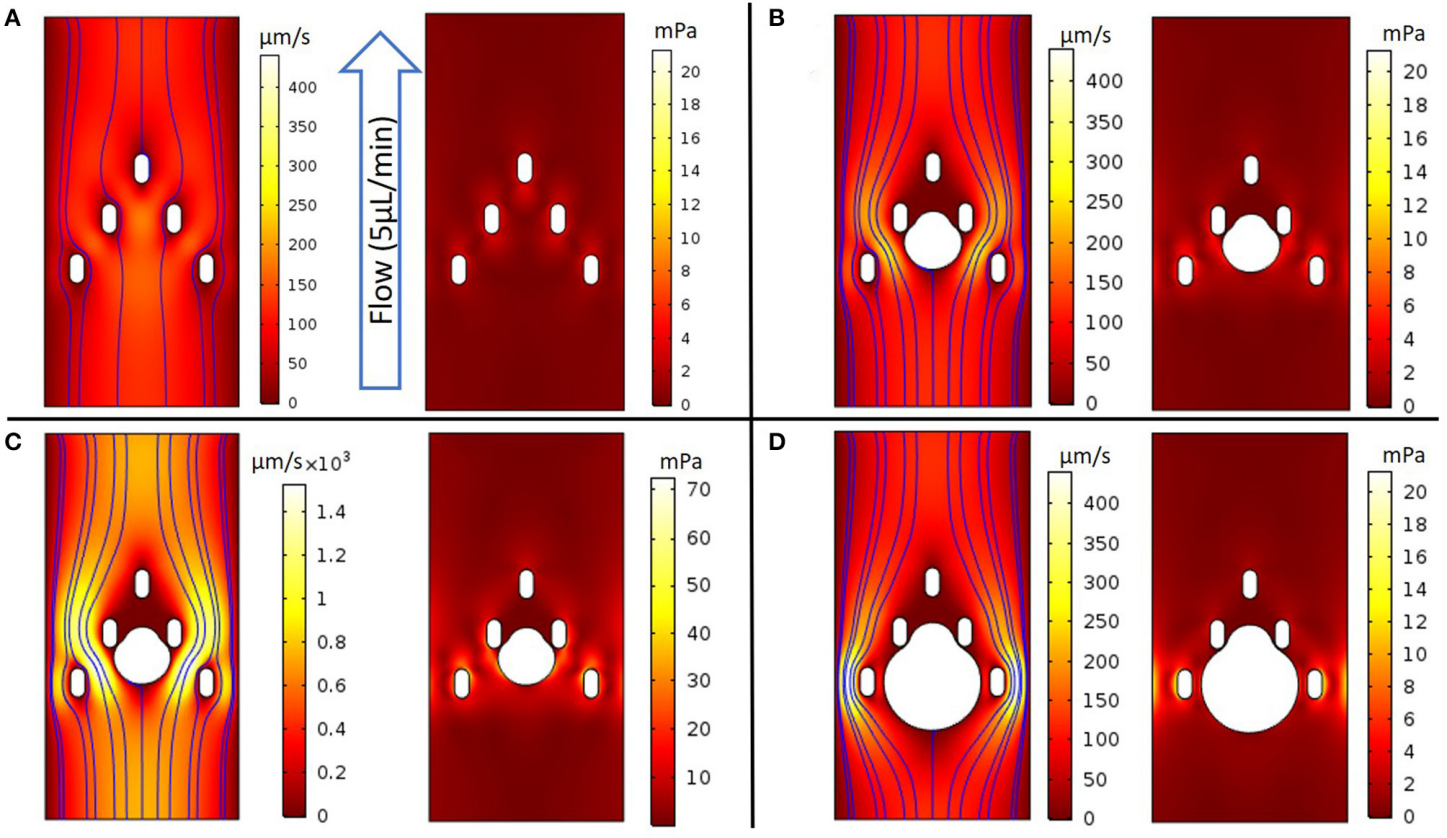
COMSOL models evaluating fluidic properties around the device's capture geometry. **(A)** (Left panel) 2D colorized analysis of velocity intensity with streamlines, (Right panel) 2D colorized analysis of shear stress without a spheroid; **(B-D)** (Left panel) 2D colorized analysis of velocity intensity and (Right panel) 2D colorized analysis of shear stress with spheroids of diameter as noted. **(B)** Capture geometry with a 300 μm diameter spheroid perfused at a 5 μL/min flow rate; **(C)** The flow rate was increased until the healthy threshold limit of 0.04 Pa was observed at a 30 μL/min flow rate; **(D)** Capture geometry with a 500 μm diameter spheroid at a 5 μL/min flow rate.

Fluid flows and shear stress within the capture region of the microfluidic device were simulated using finite element modeling (COMSOL), based on laminar flow with no-slip boundary conditions at the channel walls. For an unoccupied chamber with a flow rate of 5 μL/min, the fluid flow was fastest between the three top pillars, near the center of the channel (180 μm/s) (Figure 3A). Increased flow velocities were predicted in the center of the channel between the bottom two pillars (150 μm/s), decreasing toward the outer walls. For this geometry, characteristic shear stresses of 4 mPa adjacent to the pillars were predicted by computational modeling at the 5 μL/min flow rate.

When a 300 μm diameter spheroid was placed between the middle two pillars and exposed to the flow rate of 5 μL/min, the model predicted the highest velocities on either side of the spheroid, between the outermost middle and bottom pillars (200 μm/s) (Figure 3B). The corresponding shear stress near the spheroid reached a maximum value of 5 mPa, which we estimated to be below the 40 mPa threshold that literature reports suggest may damage tumor tissues (31). Indeed, much higher flow rates of 30 μL/min were required to achieve local shear stresses of 40 mPa at the spheroid surface, with a similar spatial distribution of fluid flow and shear stress throughout the device (Figure 3C). Finally, we modeled a larger, 500 μm spheroid that would occlude much of the channel. Interestingly, the flow was maximized at the channel periphery, away from the spheroid and beyond the outermost pillars (430 μm/s). In this limit, the highest shear stresses occurred near the bottom pillars (19 mPa), and remained below the 40 mPa threshold around the spheroid (Figure 3D). Furthermore, we modeled the oxygen consumption relative to convection and diffusion at varying flow rates, based on a volumetric consumption rate of −1.48 × 10^−6^ mol/cm^2^/s (Supplementary Figure 3), as reported in literature (32, 33). For the 5 μL/min flow rate in these experiments, the average flow velocity was 61 μm/s, which should supply a convective flux of oxygen well in excess of the total spheroid consumption, in good agreement with past reports (32, 33). At a slower flow rate of 1 μL/min, the average flow velocity was 12 μm/s, which had an appreciable effect on simulated oxygen depletion around the spheroid periphery (Supplementary Figure 3). This is qualitatively consistent with scaling arguments using dimensionless groups, where the decreased convective flux below ~10 μm/s becomes comparable to the diffusive and reactive flux, respectively (Supplementary Figures 4, 5).

### Fluorescence Imaging of Perfused Spheroids Over Time

**Figure 4:**
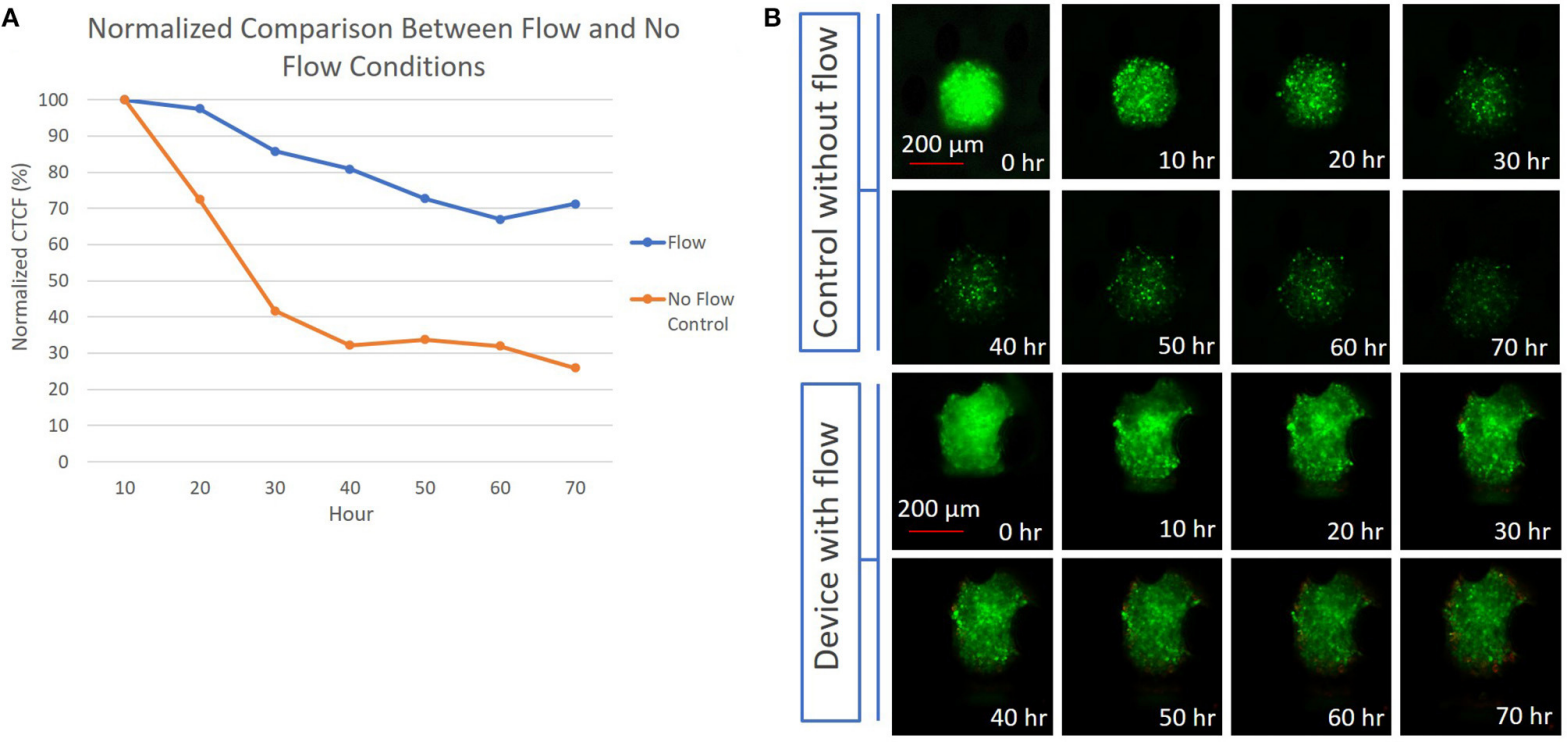
**(A)** Graph depicting the difference between the normalized CTCF values associated with the flow and no-flow control conditions over the course of 70 h; **(B)** (Top) 300 μm diameter CT26 spheroid in a device without flow, with images showing a reduction in fluorescent intensity presumed to be due to cell death over 70 h. (Bottom) 300 μm diameter CT26 spheroid in a device with perfusion flow, showing a less significant decrease in fluorescent intensity over 70 h.

As an initial proof of concept, multicellular spheroids were prepared (average diameter of 375 μm and a standard deviation of 8 μm) using CT26 colon carcinoma cells, labeled with CellTracker Green, captured in the device, then imaged in the absence of fluid flow or with perfusion (constant flow rate of 5 μL/min) over 70 h with media containing APC Annexin V. In the absence of fluid flow, the normalized fluorescence intensity of the spheroid decreased by 70% over 40 h, reaching a plateau that remained consistent from the 40 to 70 h time points (Figure 4 and Supplementary Figure 2). With perfusion, the normalized fluorescence intensity of the spheroid only decreased by 30% over 60 h, reaching a plateau at the 60 h time point. Overall, the CTCF values were higher in the center of the spheroids compared to the edges. The flow and no-flow control curves were normalized at the 10 h time point rather than at 0 h due to an anomalous light exposure issue for both conditions at time *t* = 0. Normalization of the data at the 10 h time point likely understates the difference between the flow and no-flow control conditions, due to the steepness of the no-flow curve at this point. Further analysis involving perfusion and permeation of a live-dead stain such as Annexin V will be required to further validate extended viability of captured spheroids in this system.

## Discussion

We report a microfluidic device to capture and culture multicellular spheroids under dynamic perfusion over periods of several days. This monolithic structure was constructed solely *via* 3D printing with multiscale vertical features, including arrays of posts for spheroid capture, bubble traps, and tubing connectors. The manufacturing time and scalability of the device presented here surpasses that of existing platforms (20, 21). Specifically, the device can be produced and ready for use in an hour in a half, compared to a time frame of several weeks for traditional microfluidic platforms constructed using photolithographically defined master molds. The optimized 3D printing parameters, simulated flow conditions, and modular design allow for rapid iterations to adapt to various sample sizes while maintaining device integrity and flexibility for testing configuration. Both the feature geometry, in this case post-diameter and spacing, and the height of the microchannel, can be adjusted to accommodate larger spheroids. As the spheroid size, feature sizes and channel dimensions are varied, the flow rate can be adjusted to accommodate the oxygenation requirements and targeted shear range for a specific application.

Initial perfusion studies suggest that this device, under dynamic perfusion, has the potential of extending tumor spheroid viability compared with static culture conditions. These tests, along with the simplified design and short production time, position this platform as promising development in oncology drug testing platforms. This advance is particularly relevant to immunotherapy-based testing, as static culture devices cannot fully simulate the *in vivo*-like immune microenvironment. Future work will expand the device's capability to further expand the viability of biological samples and explore retrieval of samples from the device for further testing. Ultimately, this device will enable researchers and clinicians to test various immunotherapies in a scalable, rapid, and *in vivo*-relevant manner.

## Data Availability Statement

The raw data supporting the conclusions of this article will be made available by the authors, without undue reservation.

## Author Contributions

AM, IW, and JB designed research. AM performed experiments. AM analyzed data with feedback from IW and JB. All authors wrote the paper and approved the final submission.

## Conflict of Interest

JB was a co-inventor on patent applications that address aspects of the microfluidic technology reported here; those patent applications are assigned to Draper. The remaining authors declare that the research was conducted in the absence of any commercial or financial relationships that could be construed as a potential conflict of interest.
