# Supplementary material for: 3D Printed Monolithic Device for the Microfluidic Capture, Perfusion, and Analysis of Multicellular Spheroids
Source: Front Med Technol. 2021 Apr 15;3:646441. doi: 10.3389/fmedt.2021.646441 (PMC8757790; doi:10.3389/fmedt.2021.646441)
Supplement: Supplementary file 1 [file Data_Sheet_1.pdf]

Supplementary Material

1 Supplementary Data

1.1 3D Printing Resolution Testing

The Asiga Max X27 was used for all the light-directed 3D printed devices presented here. This printer uses a high power ultraviolet light emitting diode (385 nm wavelength) with digital light processing and has a pixel resolution of 27 µm by 27 µm. Computer aided designs were prepared in SolidWorks 2016 (Dassault Systems, Waltham MA) as an .STL file at highest resolution (“fine”), then imported into Asiga Composer to optimize layer thickness and build orientation. Three types of test structures were printed to optimize feature resolution. First, layer thickness was assessed using flat steps with 10 µm height intervals. Second, cylindrical posts were designed with diameters ranging from 27 µm to 270 µm at two heights of 125 µm and 250 µm. Third, channels with rectangular cross sections were designed with sizes ranging from 650 µm wide by 125 µm high to 1 mm by 1 mm.

Prior to each print, the vat was cleaned and filled with resin to a height of 10 mm. Print conditions were systematically evaluated at different settings for control of layer thickness (thicknesses ranging from 0.01 – 0.1 mm), cure-through multiplier (0.1- 2), and offset value (0.01 – 0.6 mm). The build was then commenced and would complete the device in around 45 minutes. Using a specialized flat razor, the build was removed by wedging the razor between it and the build platform while preparing to catch it with a free hand. Once removed the device was post-treated by a three step cleaning procedure consisting of a 5 minute clean in a “dirty” bath of 97% isopropanol, a 5 minute clean in a “clean” bath of 97% isopropanol, and then drying with an air gun. Finally, printed structures were imaged using a scanning white light interferometer (Keyence VR-3100, Osaka, Japan), and analyzed using the instrument software (Keyence).

Microfluidic devices were 3D printed similarly to the test structures, followed by three additional steps. First, the channel was further cleaned by running 97% isopropanol into the channel via a syringe connected to the outlet port. Second, a series of 3600, 4000, and 6000 grit sandpapers was used sequentially (~1 minute each) to polish the capture region to improve imaging clarity and quality. Finally, a post-cure in nitrogen gas for 3 minutes completely cross-linked the resin, finalizing it for use with biological samples.

1.2 Cell Culture and Spheroid Formation

CT26 cells were subcultured from 1:4 to 1:10 ratios with media replacement every 2-3 days, based on the recommended ATCC protocol. Prior to seeding, CT26 cells were trypsinized and stained with CellTracker Green CMFDA Dye (Invitrogen C7025) at a concentration of 20 µM in PBS for 15 minutes, then resuspended in media to a cell density of at least $1x10^6$ / mL.

Molten agarose was cast against the 3D Petri Dish elastomeric masters, then allowed to cool and solidify. The cooled agarose molds were placed in a 12 well plate and prepared for the introduction of cells by soaking in 2.5 mL complete growth media twice for 15 minutes. To seed 200 µm diameter spheroids, the 24-96 3D Petri Dish geometry was used with a 75 µL droplet of containing 96,000 cells. To seed 300 µm or 500 µm diameter spheroids, the 24-35 3D Petri Dish geometry was used with a 75

µL droplet of containing 118,000 and 547,000 cells, respectively. The droplet was gently pipetted down while moving the tip across the agarose surface to uniformly distribute cells and remove any air bubbles. The single cell dispersion then aggregated via cell-cell adhesions over 48 h. Spheroids were removed from the 3D Petri Dish by inverting the 3D Petri Dish and centrifuging for 5 minutes at 500 rpm (Thermo Sorvall Legend XT with a TX-1000 bucket rotor.)

1.3 Microfluidic Device Setup

A 5 mL syringe with a gauge 12 blunt tip was used to manipulate the biological samples for transfer into the device's bubble trap port. The device was filled with complete media and the bubble trap port was vented to remove any bubbles. A stopper screw was also placed on the inlet after filling the device with media to prepare for spheroid transfer. The 5 mL syringe was partially filled to about 2 mL with complete media before attempting to remove the spheroids from the 12 well plate. The extra media was used to promote transfer of the sample into the device's channel. The injection was done slowly by hand by dipping the syringe into the 12 well plate and visually choosing a spheroid. To avoid breaking the sample apart as a result of excessive flow and shear rates, the syringe was dipped into the well about 2 mm from the sample and sucked into the syringe just until the sample was not visible. Removal of the syringe also must be done slowly and at an angle such that the syringe is parallel to the floor, to prevent dislodgment of the spheroid from the tip of the syringe. Transfer into the channel was done by gently pushing the syringe's plunger with the syringe inside the bubble trap and angled towards the capture geometry. Validation of sample capture was completed using a fluorescence microscope for visual verification. If the spheroid was not in the capture geometry but further up in the channel, tilting the device to 90˚ with respect to the ground and tapping the device against a surface often facilitated the spheroid to fall into the capture geometry.

1.4 COMSOL Oxygen Model

A 3D model of the capture region holding a 300 µm diameter spheroid was created in SolidWorks 2016 and imported into COMSOL. A new work plate was cut through the center of the channel to produce a 2D representation for analysis. All space within the model aside from the posts, walls, and spheroid were defined as water as a comparable medium to media within the model. A laminar flow study was chosen to provide flow based metrics to the model while a transport of diluted species study was chosen to provide the oxygen consumption value from the spheroid. The walls of the channel and posts were defined as no slip conditions while the inlet provided flow rates of 5 µL/min, 2.5 µL/min, and 0.001 µL/min with both an entrance length and thickness of 1 mm. For the transport of diluted species, the diffusion coefficient (D_i) was defined as 2e-9 m^2/s and evaluated as a stationary study using the following equations (32).

$$(2)\ \nabla \cdot (-D_i \nabla c_i) + u \cdot \nabla c_i = R_i$$

$$(3)\ N_i = -D_i \nabla c_i + u c_i$$

Inflow of oxygen originates from the entry of the channel at a concentration (c) of 0.205 mol/m^3 as a concentration constraint boundary condition type. The boundary of the spheroid was defined as the region of flux with a general inward flux ($N_{0,c}$) of -1.48e-6 $mol/(m^2*s)$ solved by the following equation (33).

$$(4) -n \cdot N_i = N_{0,i}$$

The major output of this study is a 2D surface plot of the transport of diluted species depicting the concentration of oxygen throughout the channel in response to flow and the spheroid's consumption rate.

1.5 Scaling Analysis

To corroborate the COMSOL simulations, we performed the following scaling analysis. First, the relative contributions of axial convection and lateral diffusion were compared using the Peclet Number $Pe_W = \frac{\bar{U}W}{D}$, where $\bar{U}$ is the average fluid velocity in the channel, W = 0.1 cm is the channel width, and D = $2 \cdot 10^{-5}$ cm^2/s in the diffusion coefficient of oxygen in water at 25°C (32, 33). The convective and diffusive timescales are comparable when Pe ~ O(1), which corresponds to $\bar{U} = 2\ \mu m/s$. Thus, convection should dominate over diffusion when Pe >> 1, and $\bar{U} \gg 2\ \mu m/s$. (Figure S4).

Next, the relative contributions of convective and reactive flux were compared using a mass balance on a spheroid of radius $a = 150\ \mu m$, consuming oxygen with a volumetric uptake rate $R_{O2} = -3 \cdot 10^{-8}$ $mol/cm^3/s$ (assuming maximum oxygen consumption rate where Michaelis-Menten kinetics are saturated):

$$(5) \quad \bar{U} C_{O2} (4\pi a^2) \sim R_{O2} \left(\frac{4}{3}\pi a^3\right)$$

where the oxygen concentration in media C_{O2} = 0.2 mM = $2 \cdot 10^{-7}$ moles / cm^3 (32, 33). Thus, when $\bar{U} = 7.5\ \mu m/s$, the nondimensional parameter $R_{O2}(a)/3\bar{U}C_{O2}$ ~ O(1), so that the convective flux is balanced by the reactive flux. Note that nondimensional parameter is analogous to a Damköhler number. Convection should dominate over reaction when $\frac{R_{O2}(a)}{3\bar{U}C_{O2}} \ll 1$ and $\bar{U} \gg 7.5\ \mu m/s$. (Figure S5). For comparison, a larger spheroid of radius $a = 250\ \mu m$ requires a faster average fluid velocity $\bar{U} = 13\ \mu m/s$ to roughly balance convective and reactive flux. Based on this scaling analysis, our experimental perfusion of spheroids with average flow speed of 61 *μm/s* should be more than adequate to match oxygen consumption, which is also qualitatively consistent with the computational models, which range down to 12 *μm/s*.

2 Supplementary Figures and Tables

2.1 Supplementary Figures

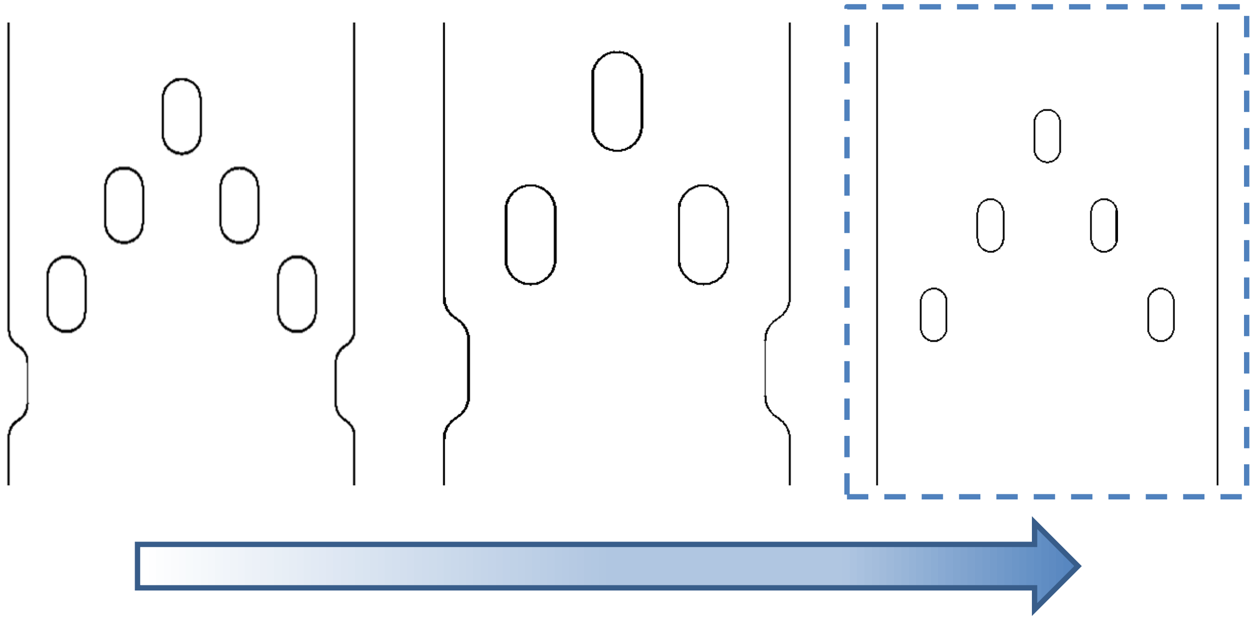

Supplementary Figure 1. Example of the progression of revisions to the capture feature design, with the final feature geometry in the channel shown in a dashed frame on the far right.

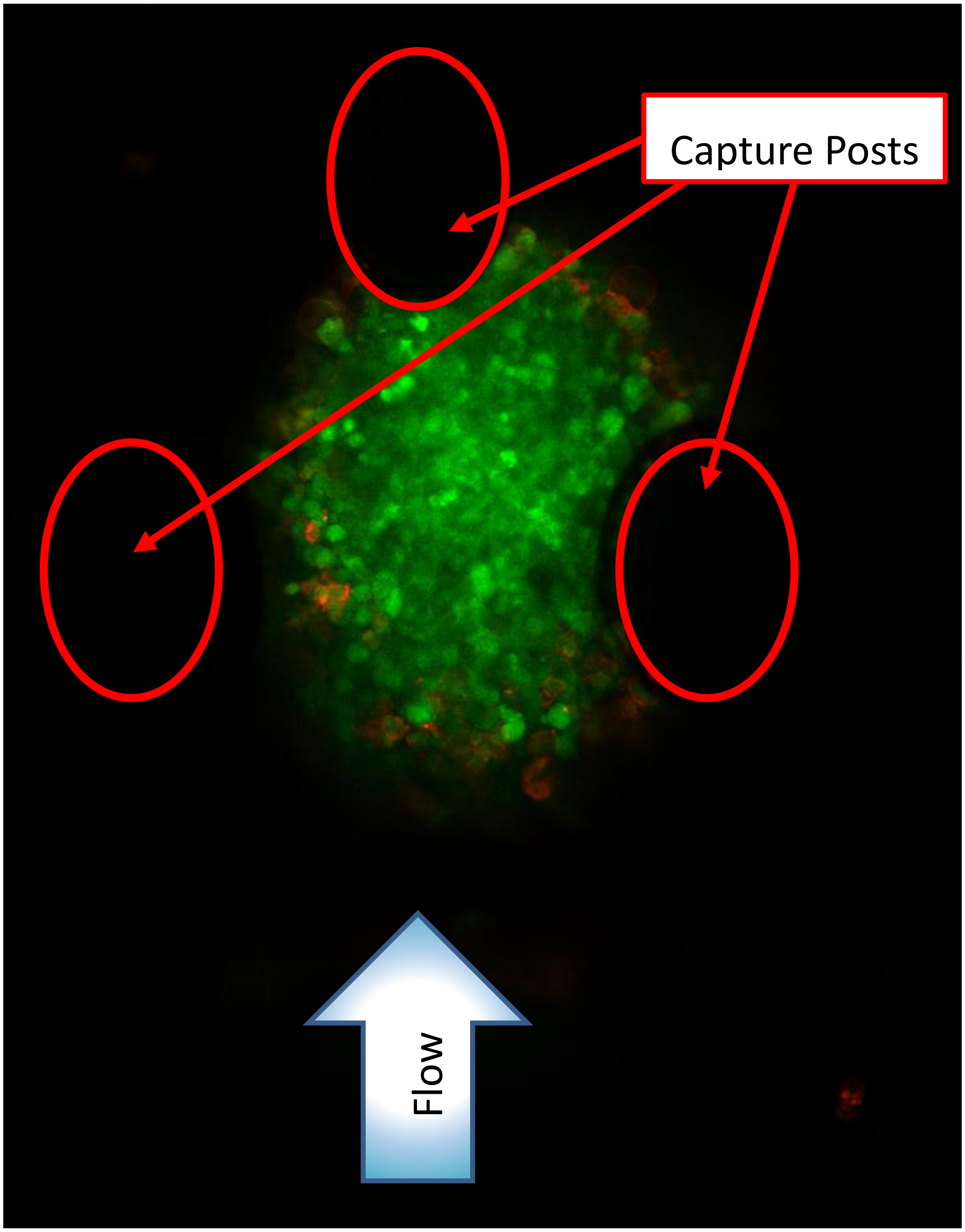

Supplementary Figure 2. Positioning of the CT26 spheroid with respect to the capture posts and direction of flow.

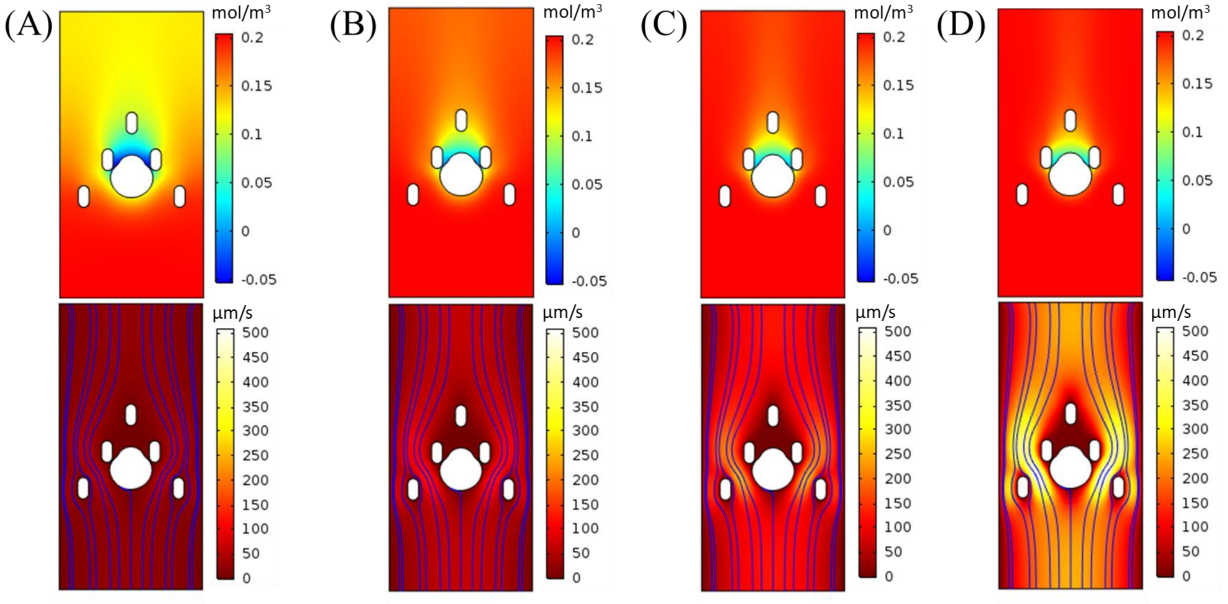

Supplemental Figure 3. COMSOL models evaluating oxygen consumption by 300 µm spheroid in the device's capture geometry with corresponding velocity plots below; A) 1 µL/min flow showing insufficient perfusion of oxygen to the spheroid (average velocity 12 µm/s); B) 2.5 µL/min flow showing limited perfusion of oxygen to the spheroid (average velocity 30 µm/s); C) 5 µL/min flow showing sufficient perfusion of oxygen to the spheroid to limit hypoxic regions (average velocity 61 m/s); D) 10 µL/min flow showing sufficient perfusion of oxygen to the spheroid to limit hypoxic regions (average velocity 122 µm/s).

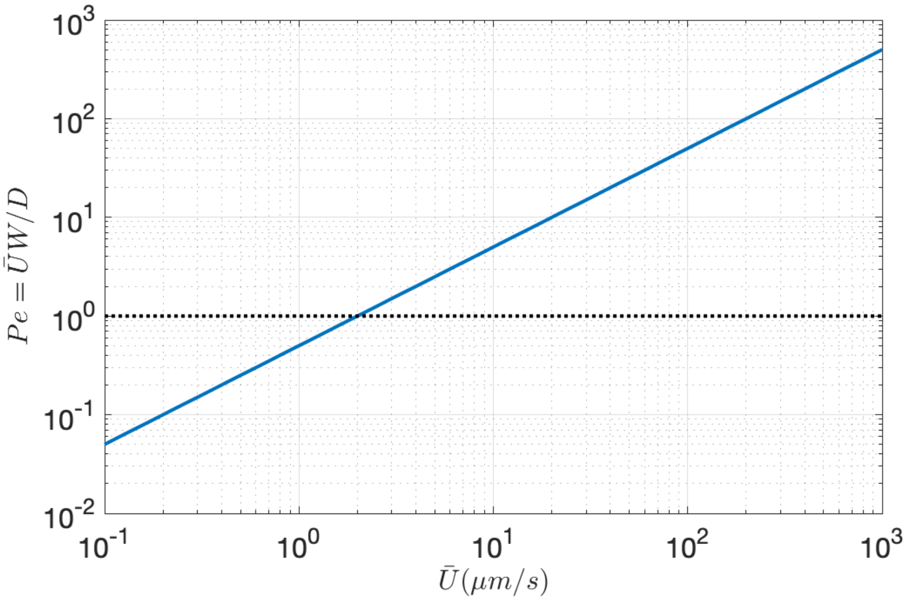

Figure S4. Comparison of Peclet number $Pe_W = \bar{U}W/D$ vs. average channel velocity $\bar{U}$. For this channel geometry, the width W = 0.1 cm and the diffusion coefficient of oxygen in water at 25°C is D = $2 \cdot 10^{-5}$ cm^2/s.

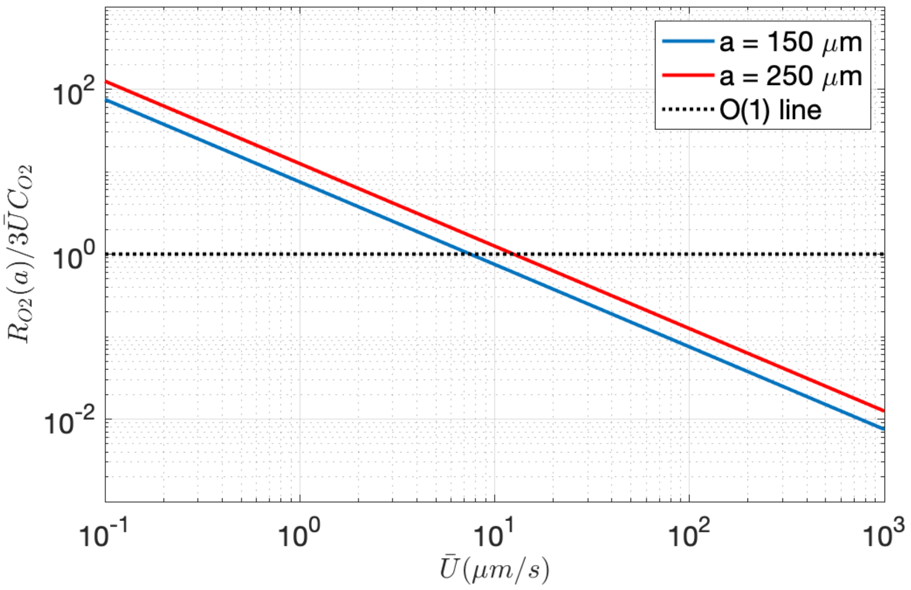

Figure S5. Comparison of convection and reaction using nondimensional parameter $R_{O2}(a)/3\bar{U}C_{O2}$ vs. average channel velocity $\bar{U}$, for spheroid radius a = 150 μm (blue) and a = 250 μm (red). The volumetric uptake rate is $R_{O2} = -3 \cdot 10^{-8}$ $mol/cm^3/s$ (assuming maximum oxygen consumption rate where Michaelis-Menten kinetics are saturated) and the oxygen concentration in media C_{O2} = 0.2 mM = $2 \cdot 10^{-7}$ moles / cm^3.
